# Enantiomorphic normalization of focally lesioned brains

**DOI:** 10.1016/j.neuroimage.2007.10.002

**Published:** 2008-02-01

**Authors:** Parashkev Nachev, Elizabeth Coulthard, H. Rolf Jäger, Christopher Kennard, Masud Husain

**Affiliations:** a Department of Clinical Neuroscience, Imperial College London, UK; b Institute of Cognitive Neuroscience, UCL, London, UK; c Institute of Neurology, UCL, London, UK

**Keywords:** MRI, Focal brain damage, Spatial registration

## Abstract

In order to make spatial inferences about the brain across a group of patients, it is usually necessary to employ some means of bringing each brain image into register with either a group mean image or a standard template. In the presence of focal brain lesions, automated methods for performing such so-called normalization are liable to distortion from the abnormal signal within the lesion, especially when the non-linear warping necessary for maximum registration fidelity is used. The most frequently used method for minimizing this distortion – cost function masking – simply eliminates the lesioned area when deriving the normalization parameters. As lesion size increases, however, the normalization error may be expected to rise steeply since the volume of brain from which the parameters are derived falls with it. Here we propose an alternative non-linear registration method that exploits a natural redundancy in the brain – the enantiomorphic relation between the two hemispheres – to correct the signal within the lesion using information from the undamaged homologous region within the contralesional hemisphere. As lesion size increases, the normalization error should theoretically asymptote to inter-hemispheric differences, which are both quantifiable and much lower than the inter-subject difference. Using SPM’s non-linear normalization routines, we evaluate this technique with images of normal brains to which lesions selected from a large dataset have been artificially applied. Our results show the enantiomorphic method to be vastly superior to cost function masking across subjects, lesion characteristics, and brain voxels. We therefore propose that it should be the method of choice for normalizing images of focally lesioned brains.

## Introduction

It is impossible to make valid comparisons between the brains of different subjects, for the purposes of structural analysis or the spatial localization of functional data, without some means of matching homologous areas between them. This process – termed spatial normalization – is commonly performed by transforming each source brain image so that it matches a standard brain template. The dominant method for estimating the necessary transformation is by finding the deformation parameters that minimize the mismatch between the source and the template image voxel intensities (e.g., Collins et al., 1994; Friston et al., 1995; Jenkinson and Smith, 2001; Woods et al., 1992). In the most sophisticated of these algorithms, an initial relatively crude, linear (affine) transformation, is followed by a finer, non-linear transformation that allows close matching of image detail. It has been shown that the second, non-linear step, substantially improves the quality of registration (Ashburner and Friston, 1999; Brett et al., 2001).

Although these algorithms have been shown to produce excellent results when applied to normal brains, a problem arises if the source images contain areas of signal abnormality such as are present in patients with focal brain damage. Since the algorithm seeks to minimize the voxel intensity mismatch across the whole brain, rather than select landmarks, a localized area of abnormal signal in the source image will distort the transformation. Affine-only transformations are less susceptible to such effects, as an attempt to minimize the differences between the lesion and the template will result in substantial mismatching elsewhere in the brain.

There are five general ways to minimize the impact of this problem. The first is to use a purely affine registration and accept a poorer image match. The second is to use a manual method, where the transformation is calculated on the basis of homologous landmarks identified by hand: this is laborious, time consuming, and operator-dependent (Damasio and Damasio, 1989; Frank et al., 1997; Mazzocchi and Vignolo, 1978). The third is to apply a homologous “lesion” to the template image; this, however, requires that the experimenter knows where the lesion is on the template, which is often the reason for the normalization in the first place. The fourth involves normalizing a fusion image composed of the mean of the source scan and its mirror image, thereby potentially extending the effect of the abnormality to the contralateral hemisphere (Weiller et al., 1995). The fifth, and most widely used, method simply ignores the area of the lesion and its immediate vicinity when calculating the parameters and applies the resultant affine and non-linear transformations in the standard fashion: a technique known as normalization with cost function masking (Brett et al., 2001). Current implementations of this method do not automatically determine the location of the lesion – which would be difficult to achieve given the heterogeneity of lesion signal intensities relative to the rest of the brain – and require the user to supply an additional, binary mask image corresponding to the location of the lesion.

The last approach has been shown to be superior both to purely affine and unmasked non-linear registration (Brett et al., 2001). However, it is open to an obvious criticism. As lesion size increases, the volume of brain used to estimate the registration parameters decreases. To the extent to which the area of the brain masked impacts on the registration process, it is therefore reasonable to expect a monotonic relation between lesion size and quality of registration that will generally asymptote to the inter-subject difference or worse, i.e., no registration at all.

If lesion size varied uniformly across the brain such an effect would merely add noise. However, if it does not – as is the case for vascular lesions (Husain and Nachev, 2007) – there is a risk that the registration of different parts of the brain will vary in quality depending on their correlation with lesion size, thereby introducing a spatial confound. The issue is potentially more critical than simply the general quality of the data: in these circumstances the normalization process may bias any spatial inferences drawn from it.

We therefore propose a new automated method for normalizing lesioned brains that exploits two fundamental characteristics of the brain imaging data: first, that the brain is symmetrical or, to put it more precisely, the hemispheres are enantiomorphically related; and second, that focal lesions generally do not cross the midline. Rather than masking out damaged areas our method replaces them opto-isometrically (i.e., volume mirrored) with signal from the homologous region of the undamaged contralateral hemisphere, and then proceeds to perform a standard SPM normalization. Thus the lesioned area is corrected on the basis of the best available guide to its appearance before it was damaged: its contralateral counterpart. Theoretically, as lesion size increases our method should asymptote to the error resulting from inter-hemispheric differences, which can reasonably be expected to be substantially lower than the inter-subject difference (Thompson et al., 1996; Watkins et al., 2001). Furthermore, since maps of brain asymmetry exist (Toga, 2003), it is possible to address the confounding effects of this error explicitly at the inference stage.

Here we extensively test this theoretical conjecture by comparing what might be called an enantiomorphic method to normalization with and without cost function masking, using a large dataset of normal brain images to which lesions have been artificially applied.

## Methods

### Algorithm

**Fig. 1:**
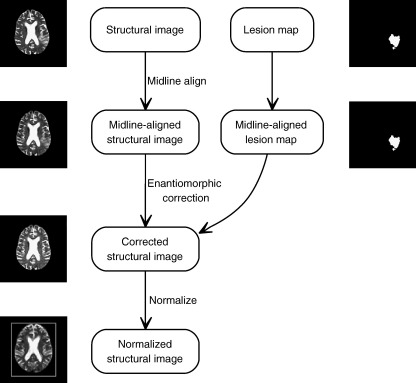
The enantiomorphic normalization algorithm. A structural image and a matching lesion mask are aligned in the sagittal midline using rigid body coregistration to the opto-isometrically flipped version of the structural image. The mask is then flipped opto-isometrically and used to extract the signal within the precise region in the unaffected hemisphere that corresponds to the lesion on the other side. This is then used to replace the signal corresponding to the lesion. The resultant corrected image is then normalized using SPM2's standard spatial normalization routine.

The steps are outlined in Fig. 1. We assume as a starting point a structural image and a binary lesion mask in the same stereotactic space. The lesion mask may be generated by manually tracing the lesion on the structural image, or via some automated algorithm: the precise means are immaterial to our method.

The first step is to align the structural image in the sagittal midline so that homologous regions in the two hemispheres may be matched in the subsequent steps. This can be achieved simply by deriving the parameters for a rigid body coregistration of the structural image and its opto-isometric version using SPM2’s coregistration routine, and applying to the structural and lesion images the appropriate rotations half-way. Next the midline-aligned lesion mask is inverted opto-isometrically and used to extract the corresponding region within the unaffected hemisphere of the structural scan. The signal within the lesioned area of the midline-aligned structural image is then replaced with the extracted signal. This leaves a midline-aligned structural image with the lesioned area opto-isometrically replaced by signal from the contralateral hemisphere. The final step is to normalize this image using SPM2’s non-linear normalization routine, or whatever normalization routine the user prefers.

Note that the initial, rigid-body coregistration step will inevitably be susceptible to some distortion by the lesion, however – as we have argued already – this distortion will be minimal since a focal lesion-induced mismatch will be countered by large mismatches elsewhere. More importantly, the quality of registration obtained in this step directly affects only the accuracy of the enantiomorphic correction, not the final normalization itself. There is therefore no need to quantify any error introduced by this step independently of the whole process.

### Evaluation

There is no truly objective way of determining how well a given brain has been normalized. However, if we artificially apply a lesion to a normal brain image and compare the transformation parameters derived from normalizing the lesioned and unlesioned versions of the image, we can make an objective assessment of how well the normalization algorithm copes with the impact of the lesion (Brett et al., 2001).

The normalization parameters can be explicitly compared by generating the deformation field they describe. A deformation field is a volume image of the same dimensions as the template that maps the estimated displacement necessary to bring each voxel into alignment with its homologue in the source image. Similar deformation fields will naturally have similar patterns of voxel displacement. The precise extent of similarity between two deformation fields can be quantified by computing the displacement of one field relative to another, on a voxel by voxel basis. This is simply the square root of the summed squared differences in each direction (*x*, *y*, and *z*), for each homologous pair of voxels in the two deformation fields being compared. The result is a volume map of the displacement of one deformation field relative to another: what might be called a *displacement* field.

In order to obtain a whole brain measure of similarity we then calculate the root mean square (RMS) of the displacement field (Brett et al., 2001), averaging across all voxels that fall within the brain as defined by the template brain image.^1^ We can also use the displacement fields to do voxel-wise comparisons across subjects so as to identify any consistent spatial differences in the quality of registration.

**Fig. 2:**
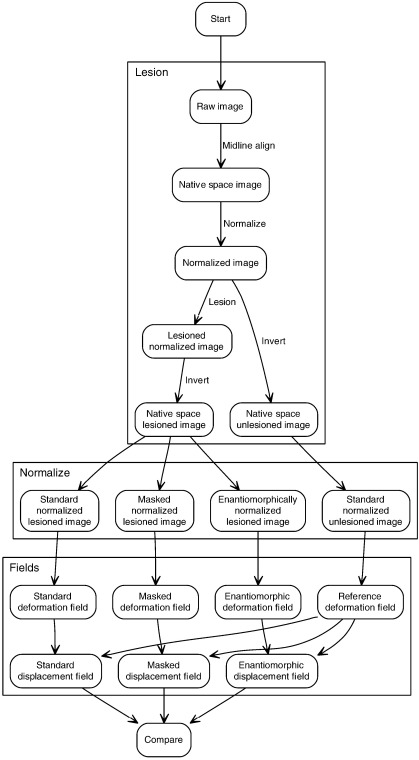
Evaluation procedure. A set of 305 lesions was applied to each of a set of normal brain images, and the resultant artificially lesioned volumes together with the normal images were normalized in three ways: with and without cost function masking, and with the enantiomorphic method. The normalization parameters in each case were used to generate a set of deformation fields, from which displacement fields relative to the normalization of the normal brain images were computed. The displacement fields thus provided an index of the normalization error introduced by the lesion for each method, lesion and subject, thereby allowing us to determine which method minimized the error to the greatest extent. See main text for details.

The experiment therefore consisted of the following steps (Fig. 2). We first took two sets of brain images of different subjects (*n*1 = 50, *n*2 = 42) free of any focal lesions and used a large independent dataset of brain lesions (*n* = 305) to generate artificially lesioned versions of each image (i.e., a total of 50 × 305 = 15250 lesioned volumes for the first set, 12810 for the second set). This was done so that we could make comparisons between the lesioned and unlesioned versions of the same image, thereby isolating the specific effect of each lesion on the normalization process. Second, we normalized both versions of each image using SPM2’s standard normalization routine with and without cost function masking, and with the enantiomorphic method. Third, each set of normalization parameters was used to generate a corresponding deformation field. Fourth, the deformation fields from the lesioned versions of each image were subtracted from the deformation field derived from the unlesioned version of the same image – as described above – to generate a set of displacement fields, for each subject, lesion, and method. Finally, we performed a series of whole brain and voxel-wise comparisons between these displacement fields so as to determine how well each method minimized the effect of the lesion, and how this was influenced by the parameters of the lesion.

### Data

**Fig. 3:**
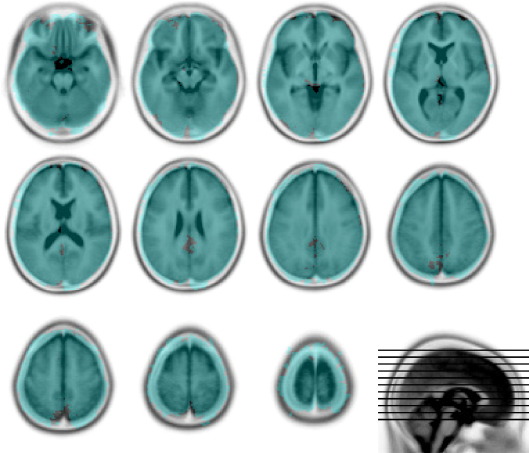
Lesion coverage. The overlay shows every voxel that is affected in at least one lesion (in blue). Note that the distribution of voxel involvement across the whole series is not even (data not shown). The underlay is the Montreal Neurological Institute 152 normal subject T2 average template (rendered in inverted grayscale) supplied with SPM (http://www.fil.ion.ucl.ac.uk/spm/). The figure was generated using MRIcro (http://www.sph.sc.edu/comd/rorden/mricro.html).

The spatial characteristics of the 305 lesions used to generated the artificially lesioned images were obtained from a large set of normalized brain images with real focal vascular lesions unselected except for the requirement that lesion volume should exceed the fairly low threshold of 5 cm^3^. They therefore vary widely in size and tend to affect certain areas more commonly than others. Nonetheless, the brain coverage (shown in Fig. 3) is comprehensive, and vastly greater than in any previous study.

In generating the artificially lesioned images, we used the spatial parameters for each lesion to set the voxels within it either to zero or – in a separate experiment for the T2 dataset – to the mean of the normal signal intensity within the area of the lesion. Although the two methods we are comparing here are largely insensitive to the lesion intensity within the bulk of the lesion, cost function masking may be affected by propagation of the lesion beyond the boundaries of the mask resulting from the smoothing step that precedes most normalization algorithms. Mean-filled lesions – being unrealistically “benign” – allow us to exclude this possibility as a reason for the difference between the two methods. Note that the comparison with mean-filled lesions is informative only in relation to this specific question. One cannot, for example, reliably use it to determine whether it is the removal of abnormal signal intensity or the restoration of the normal pattern of signal in the lesioned area that is decisive. This is so because filling an area with the mean of the signal normally found within it will variably affect – in a way that is hard to quantify – its pattern similarity to normal brain depending on the location and extent of the lesion.

A criticism that can be raised against using artificially lesioned brain images is that they might differ globally from images of real lesioned brains, i.e., that apparently normal areas in real lesioned brains might be subtly abnormal in a way that might significantly affect the fidelity of normalization in a method-dependent way. For example, it might be argued that since patients with stroke often have evidence of previous vascular damage, and such damage generally respects the midline, the brains of stroke patients may be inherently less symmetrical than those of people without cerebrovascular disease. Thus the error associated with a method that is sensitive to the symmetry between brains may be underestimated if the artificially lesioned brain images are based on the brains of entirely normal people.

To minimize this effect, the normal brain images used in the first set were derived from a clinical population: 50 individuals referred to a stroke clinic but not found to have had a stroke. Although free of obvious focal lesions, they showed somewhat greater atrophy than normal brains, and may be expected to have exhibited inter-hemispheric asymmetry similar to that found in a stroke population. These considerations clearly do not apply to focal lesions that do not generally occur in the setting of chronic disease, such as brain tumors. The data derived from these brains are therefore likely to be weighted slightly against our method. The images were clinical grade T2-weighted MRI scans of resolution 1 mm × 1 mm × 6.5 mm. Since the comparison is between different normalizations of different versions of the same structural image, it ought not to matter that we use T2 images rather than the more conventional T1. The same applies for the rather anisotropic acquisition sequence typical of clinical images. However, it may be argued that these characteristics will reduce the requirement for non-linear transformations and therefore fail to capture the effect on the component of the registration that is of greatest interest here. We therefore carried out a separate analysis with a second set of isotropic, high-resolution (1mm3), T1-weighted images derived from 42 normal subjects.

### Procedure

The experimental procedure is outlined in Fig. 2. For each dataset, we first aligned the structural images to the vertical midline by applying half the appropriate rotations estimated from a rigid body coregistration of the image to its opto-isometrically flipped version. So as to match the normalized lesions to each subject’s normal image we first normalized each normal image to the standard SPM2 T2 template. For each subject and each lesion we then set the voxels in the normal image corresponding to any part of the lesion to an intensity of zero or (for the T2 dataset) to the mean of the normal signal within the confines of the lesion. The normal volumes and the resulting artificially lesioned volumes were then transformed back into their native space by applying the inverse of the computed transform.

In the next step, each volume was normalized with SPM2’s routines in three separate ways: using the standard routine, with cost function masking, and with enantiomorphic correction. When cost function masking was used, the area masked was approximately 10% larger than the lesion. When enantiomorphic correction was used, the area corrected corresponded closely to the area of the lesion, with the edges blended in with the aid of a 1mm FWHM smoothed version of the mask. The normalization parameters were the SPM2 defaults.

Following normalization, the resultant transformation parameters were used to generate the corresponding deformation fields. For each method and each lesioned volume, the reference deformation field derived from normalizing the unlesioned version of each volume was used to calculate a displacement field as previously described. All subsequent analyses are based on these fields, which excluded voxels that fell outside the brain as determined by a binary mask derived from SPM2’s standard brain mask image thresholded at 0.5.

Since the displacement data were approximately log normal, the descriptive statistics were performed by log transforming the data first and anti-logging the results. The subsequent statistical tests compared the enantiomorphic method to cost function masking only. Whole volume statistical comparisons were performed on the raw data using one-tailed, two-sample, Kolmogorov–Smirnov tests. Voxel-wise comparisons were performed by log transforming the data and entering each volume in a one-way ANCOVA with normalization type as a factor and lesion size as a covariate.

So as to aid the visualization of possible interactions between normalization error and lesion spatial characteristics, we used a non-linear dimensionality reduction technique, Isomap, to embed the lesion data – parameterized as a series of binary volumes with dimensions equal to the number of voxels – into a two-dimensional space that preserves the high-dimensional relations between the data (Tenenbaum et al., 2000). By superimposing the statistical data on the Isomap embedding, any systematic relation between a given lesion feature and normalization error may thus be more readily visualized.

The enantiomorphic correction and whole brain statistics were performed by a custom MATLAB script. Isomap embedding was performed using the author’s own Matlab code (http://isomap.stanford.edu/). All other procedures were implemented in SPM2 (http://www.fil.ion.ucl.ac.uk/spm/) run under MATLAB 7.2 64 bit for Linux with a multithreaded, Opteron-tuned, custom BLAS by Kazushige Goto (http://www.tacc.utexas.edu/~kgoto/) on a four-core 2.6-GHz AMD Opteron custom-built machine.

## Results

**Fig. 4:**
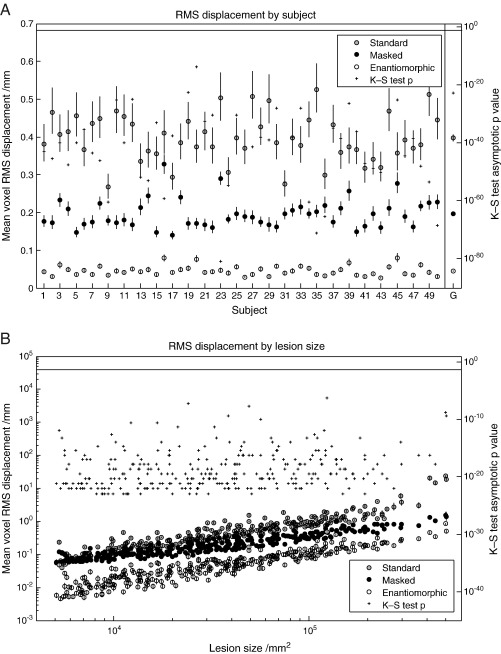
(A) RMS displacement by subject (T2 dataset with zero-filled lesions). The left hand axis shows whole brain mean voxel RMS displacement (the difference between the deformation fields describing the normalization of lesioned and unlesioned versions of the same image) with standard errors, plotted by individual subjects. The means include all 305 lesions and were derived – owing to log normality – by first log transforming the data, calculating the means and standard errors and anti-logging the results. The right hand side shows the asymptotic *p* values from a one-tailed, two sample Kolmogorov–Smirnov test carried out on the untransformed data from the masked and the enantiomorphic methods. The vertical line at the top defines the 0.05 significance level. The values in the far right column represent the group means and associated standard errors. The corresponding *p* value was derived from an appropriate Kolmogorov–Smirnov test on the group means. (B) RMS displacement by lesion size (T2 dataset with zero-filled lesions). The left hand axis shows whole brain mean voxel RMS displacement with standard errors, plotted by lesions size. The means include all 50 subjects and were derived – owing to log normality – by first log transforming the data, calculating the means and standard errors and anti-logging the results. The right hand side shows the asymptotic *p* values from a one-tailed, two sample Kolmogorov–Smirnov test carried out on the untransformed data from the masked and the enantiomorphic methods. The vertical line at the top defines the 0.05 significance level.

**Fig. 5:**
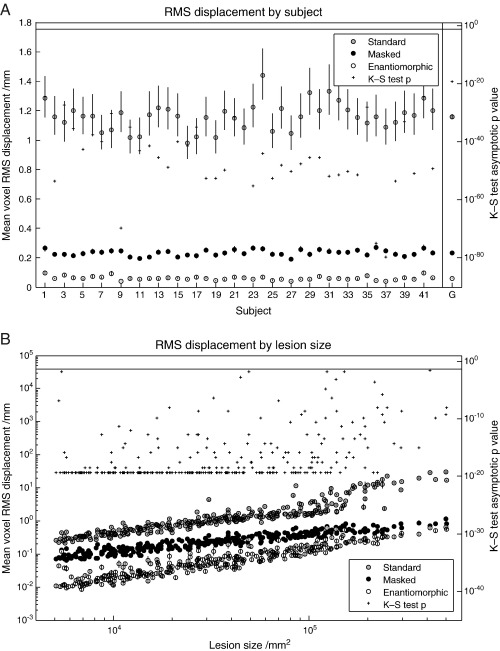
(A) RMS displacement by subject (T1 dataset with zero-filled lesions). The left hand axis shows whole brain mean voxel RMS displacement with standard errors, plotted by individual subjects. The means include all 305 lesions and were derived – owing to log normality – by first log transforming the data, calculating the means and standard errors and anti-logging the results. The right hand side shows the asymptotic *p* values from a one-tailed, two sample Kolmogorov–Smirnov test carried out on the untransformed data from the masked and the enantiomorphic methods. The vertical line at the top defines the 0.05 significance level. The values in the far right column represent the group means and associated standard errors. The corresponding *p* value was derived from an appropriate Kolmogorov–Smirnov test on the group means. (B) RMS displacement by lesion size (T1 dataset with zero-filled lesions). The left hand axis shows whole brain mean voxel RMS displacement with standard errors, plotted by lesions size. The means include all 42 subjects and were derived – owing to log normality – by first log transforming the data, calculating the means and standard errors and anti-logging the results. The right hand side shows the asymptotic *p* values from a one-tailed, two sample Kolmogorov–Smirnov test carried out on the untransformed data from the masked and the enantiomorphic methods. The vertical line at the top defines the 0.05 significance level.

**Fig. 6:**
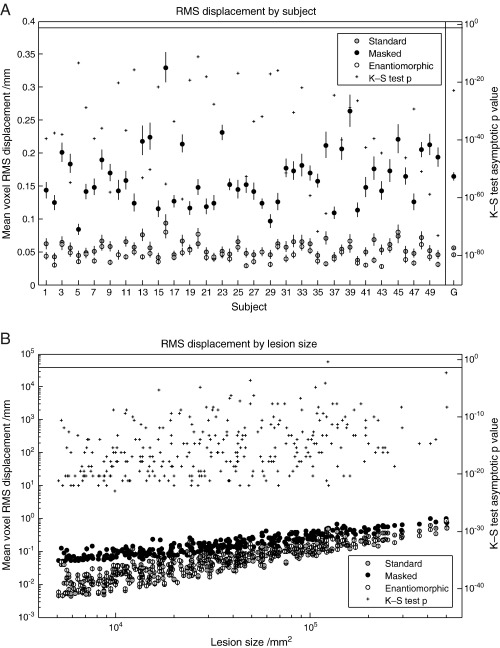
(A) RMS displacement by subject (T2 dataset with mean-filled lesions). The left hand axis shows whole brain mean voxel RMS displacement with standard errors, plotted by individual subjects. The means include all 305 lesions and were derived – owing to log normality – by first log transforming the data, calculating the means and standard errors and anti-logging the results. The right hand side shows the asymptotic *p* values from a one-tailed, two sample Kolmogorov–Smirnov test carried out on the untransformed data from the masked and the enantiomorphic methods. The vertical line at the top defines the 0.05 significance level. The values in the far right column represent the group means and associated standard errors. The corresponding *p* value was derived from an appropriate Kolmogorov–Smirnov test on the group means. (B) RMS displacement by lesion size (T2 dataset with mean-filled lesions). The left hand axis shows whole brain mean voxel RMS displacement with standard errors, plotted by lesions size. The means include all 50 subjects and were derived – owing to log normality – by first log transforming the data, calculating the means and standard errors and anti-logging the results. The right hand side shows the asymptotic *p* values from a one-tailed, two sample Kolmogorov–Smirnov test carried out on the untransformed data from the masked and the enantiomorphic methods. The vertical line at the top defines the 0.05 significance level.

The measure of interest is what we have called a *displacement field*: the difference between the deformation field derived from normalizing – using the method being tested – the lesioned version of the image, and the reference deformation field derived from normalizing the unlesioned version of the same image. We first evaluated the impact of the normalization method used for each subject, as indexed by whole brain RMS displacement across all lesions. For the T2 dataset with zero-filled lesions, the enantiomorphic method was superior to cost function masking across all subjects, on average by over a factor of four. The mean values and standard errors for each subject – owing to approximate log normality computed by log transforming the data, obtaining the statistics, and anti-logging the results – are plotted in Fig. 4A. The mean displacement across all subjects was 0.40 mm per voxel (SEM = 0.0088) for the standard method, 0.20 mm (SEM = 0.0054) for cost function masking, and 0.045 mm (SEM = 0.0018) for the enantiomorphic method. A one-tailed, two-sample Kolmogorov–Smirnov test on the untransformed data, comparing cost function masking to the enantiomorphic method, showed that the latter was superior for every subject, with asymptotic *p* values < 10^− 23^. These statistics are plotted on the right hand axis in Fig. 4A. For the T1 dataset with zero-filled lesions (Fig. 5A), the values were 1.161 mm (SEM = 0.0147) for the standard method, 0.2328 mm (SEM = 0.003) for cost function masking, and 0.0606 mm (SEM = 0.002) for the enantiomorphic method. The *p* values for all subjects were less than 10^− 24^. For the T2 dataset with mean-filled lesions (Fig. 6A), the values were 0.056 mm (0.002) for the standard method, 0.165 mm (SEM = 0.006) for cost function masking, and 0.045 (SEM = 0.002) for the enantiomorphic method. The *p* values for all subjects were less than 10^− 11^. Note that the very low error values for the standard normalization demonstrate that the mean-filled lesions were indeed unrealistically benign, and that the difference between cost function masking and the enantiomorphic methods cannot be explained by the signal intensity of the artificial lesions.

Next, we explored the effect of the characteristics of the lesion by computing analogously the mean RMS displacement for every lesion type, averaging across individual subjects. Again, the enantiomorphic method was superior to cost function masking across all 305 lesions studied, for all datasets. The results of a plot of mean RMS displacement against lesion size are shown in Figs. 4B, 5B and 6B. As might be expected, there is a rise in RMS displacement with increasing lesion size for all three methods. However, the mean RMS for the enantiomorphic method is smaller for every lesion compared with cost function masking, and this is reflected in appropriate one-tailed, two-sample Kolmogorov–Smirnov tests, whose *p* values (all except one below 0.05 significance threshold) are plotted on the right hand side axis.

**Fig. 7:**
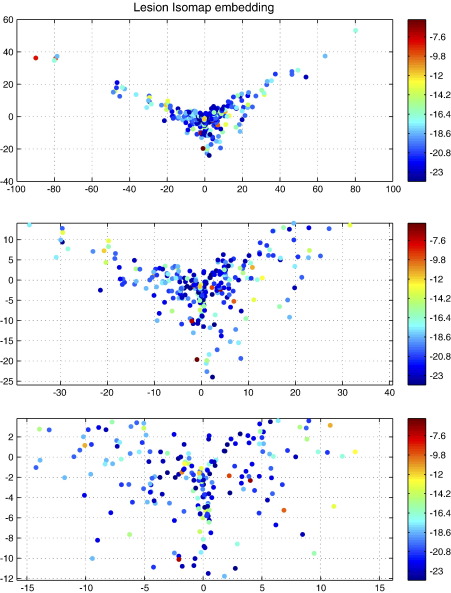
Two-dimensional Isomap embedding of the lesion data (T2 dataset with zero-filled lesions). The algorithm attempts to preserve proximity in high dimensions in the two-dimensional embedding shown here. Similar lesions are therefore likely to cluster together. The colors index the logarithm of the asymptotic *p* value from a one-tailed, two-sample Kolmogorov–Smirnov test of the difference between the cost function masking and enantiomorphic methods. Each data point represents an individual lesion. Successive plots zoom in on the central area to reveal it in more detail. The absence of a clear overlap between spatial and *p* value clustering, except for a tendency for the effects to be somewhat less significant for small lesion sizes, suggests there is no clear set of lesion features for which the enantiomorphic method is consistently inferior.

Although the enantiomorphic method was shown to be superior for every lesion tested it is possible that there is a subset of lesion features for which its superiority is more or less pronounced. Brain lesions obviously differ not only in size but also in many other dimensions, which makes it difficult to visualize a comparison across all features. We have therefore used the Isomap algorithm to reduce the dimensionality of the entire set of brain masks used to two dimensions (Tenenbaum et al., 2000). This algorithm transforms the data into an artificial low-dimensional space while seeking to preserve the intrinsic high dimensional relations between the data. Each lesion is thus placed in an artificial low-dimensional “lesion feature” space, with similar lesions being close together and dissimilar ones far apart. This mapping – with the T2, zero-filled, Kolmogorov–Smirnov test asymptotic *p* value for each lesion labelled in color – is plotted in Fig. 7. Other than for a propensity for larger lesions to show greater benefit, no decisive clustering is apparent, suggesting that there is no consistent set of lesion features for which the enantiomorphic method is strikingly more or less successful, at least on the basis of an Isomap embedding of the lesion data. Note that we do not make any hard inferences from this finding, as there was no lesion for which the enantiomorphic method is not superior.

**Fig. 8:**
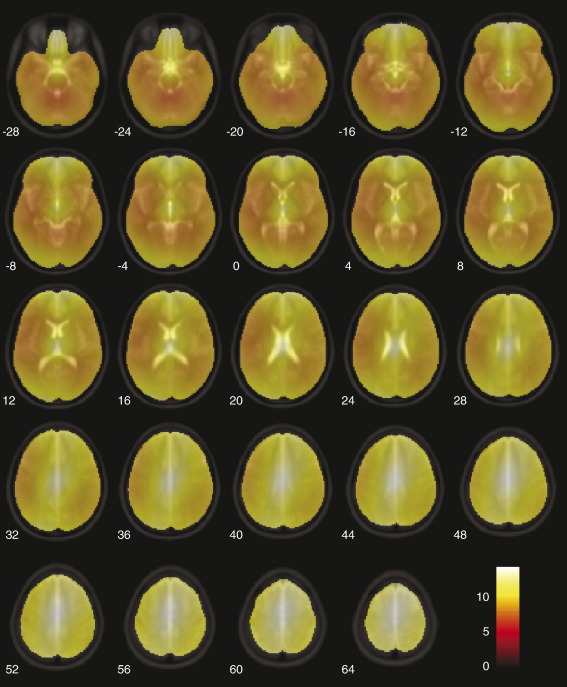
Mean SPM(t) map of the contrast between cost function masking and the enantiomorphic methods (T2 dataset with zero-filled lesions). The displacement fields for each subject (305 scans per condition) were log transformed and entered into a one-way ANCOVA with log lesion size as a covariate. Only fields derived from cost function masking and the enantiomorphic method were included in the analysis. In all subjects all voxels within the brain survived a *p* < 0.05 family-wise error corrected threshold, showing that the enantiomorphic method is superior across all voxels. The figure shows the mean *t* value across subjects. The figure was generated using the slice_overlay tool for SPM2 written by Matthew Brett (http://imaging.mrc-cbu.cam.ac.uk/imaging/DisplaySlices).

Finally, so as to assess spatial differences between the two methods on a voxel-by-voxel basis, we computed a voxel wise t map of the differences in displacement between the two test methods, using the T2 zero-filled dataset. This was done by log transforming the corresponding displacement fields for each subject and entering them into a SPM2 one-way ANCOVA with lesion size as a covariate. All voxels were above threshold for every subject. A mean map of the t contrast: “cost function masking > enantiomorphic” is shown in Fig. 8. The areas of highest significance are close to the edges of the brain, presumably because lesions not completely surrounded by normal tissue tend to be least well normalized by the masking method. It is in the normalization of cortical tissue – which is perhaps of greatest interest to imagers – that the enantiomorphic method shows the greatest advantage. The effect is somewhat attenuated in areas of the brain known to exhibit hemispheric asymmetry, but is still significantly in favor of our method.

## Discussion

The enantiomorphic method is very simple. Conceptually, it relies solely on the self-evident proposition that information from the contralateral hemisphere is generally better than no information at all. Technically, it requires only a mask image identifying the lesion site and is computationally trivial. Although we have used it in combination with SPM’s normalization routine, it is potentially applicable to other algorithms. It is readily applicable to any lesion that does not affect both hemispheres in exactly the same place.

We have presented evidence — based on a comparison of a very large number of brain images and the widest range of lesions hitherto studied in this regard — that the enantiomorphic method is superior to what is generally considered the current gold standard. This advantage is substantial and extends across subjects, lesion parameters, and voxels. We have seen that the benefit is largely indifferent to the imaging modality used – at least when clinical T2 and high-resolution T1 scans are compared – and cannot be explained merely by a difference in the extent to which abnormal signal is eliminated from the lesioned brains. Although our study is the most comprehensive of its kind and the results are unequivocal, we must make note of some potential criticisms and limitations.

First, although we have sampled a very wide range of lesion sizes we did not include lesions smaller than 5 cm^3^. This was because we did not expect that very small lesions would make a substantial difference to the quality of normalization, and such lesions would in any case be unlikely to be the subject of patient studies, which typically use patients with relatively large lesions. In any case, inspection of Fig. 4B suggests that the disparity between the two methods does not decrease with decreasing lesion size, so there is no reason to expect it to change suddenly below the threshold we have arbitrarily chosen. Nonetheless this possibility cannot be absolutely excluded.

Second, the pattern of sizes and spatial localizations of the lesions included in the analysis is derived from vascular lesions. Lesions with other spatial characteristics, such as brain tumors, may conceivably yield different results. However, the wide extent of brain coverage, and the level of statistical significance for every different lesion (given the relatively small number of subjects) would seem to make this unlikely.

Third, vascular lesions generally do not cross the midline: clearly lesions that affect the same parts of the hemisphere bilaterally would not be suitable for the enantiomorphic method. Since in such situations the lesions are not likely to be completely symmetrical, the best strategy would be to make an enantiomorphic correction for the parts that do not overlap, and use cost function masking for the remainder. An evaluation of such an approach is given in the supplementary material.

Fourth, as a consequence of the inhomogeneity in the distribution of vascular lesions certain areas such as the cerebellum and the brainstem were less well sampled than other areas. However, there is no a priori reason why they should behave differently from the rest of the brain; furthermore there was no lesion for which the enantiomorphic method did not produce better results.

Fifth, it may be that for a set of lesions heavily involving areas of most marked brain asymmetry, such as the planum temporale, the results may differ. However, the lesions used in this study covered these areas, and there was no lesion for which the enantiomorphic method was not superior. Indeed, the voxel-wise comparison showed that even in these areas the enantiomorphic method produces less error than cost function masking. Nonetheless it is impossible to exclude the possibility that there exists a lesion for which cost function masking is better: all we can say is that we could not find one in a lesion dataset vastly larger than anything previously studied in this regard.

Sixth, it may be argued that it is not the absolute size of the error, but the consistency of the spatial bias it introduces across the brain that matters most. Thus although the overall error might be lower for the enantiomorphic method, if the error consistently covaries with damage to specific areas of the brain – such as those that are associated with the greatest hemispheric asymmetry – the impact on the subsequent statistics will be more significant than for a greater degree of error that is not spatially correlated. However, as has already been pointed out, an analogous objection can be raised against the cost function masking method because different regions of the brain have different correlations with lesion size, at least for vascular lesions (Husain and Nachev, 2007). This is an unavoidable consequence of the structure of the vascular tree, and the consequent pattern of damage in stroke. A spatial bias is therefore likely to exist in any case, so what is more important is that it should be minimized—as our method clearly does better. Furthermore, any bias introduced by inter-hemispheric differences can be addressed at the inference stage with the aid of an asymmetry map (see Supplementary material). The bias resulting from location–size correlations is currently unquantified and ought to be the subject of urgent study.

Finally, it should be noted that – in common with every other normalization method currently in use – out method requires special care in the presence of brain swelling. The best strategy in these circumstances depends on the nature of the swelling. Where the swelling is local to the extent of not distorting the enantiomorphically correspondent area in the contralateral hemisphere, the lesion mask used to perform the enantiomorphic correction should cover the entirety of the swollen area. Thus both the lesion and the swelling are enantiomorphically corrected. Where the swelling is so gross that enantiomorphically correspondent area is displaced relative to its homologue our method may be no better than simply filling in the lesion with the mean of the signal within the enantiomorphically correspondent area. Inspection of Fig. 6 suggests that this may be better than cost function masking. However, we would suggest that in the presence of remote swelling it is impossible to attribute any behavioral effect to a focal structural feature in the brain – whether a lesion or something else – so the situation where our method would not be applicable is unlikely to be of great concern to most of those likely to make use of it.

## Conclusion

We have devised a simple method to aid the normalization of focally lesioned brains, and we have used a lesion dataset of unprecedented size to demonstrate its superiority across subjects, lesion parameters, and voxels. The data suggest that the enantiomorphic method ought to be the preferred way of normalizing focally lesioned brains where the area of the contralesional hemisphere corresponding the lesion is intact. Where it is so only partially, we would suggest it should be combined with cost function masking. Our method also permits the normalization of focally lesioned brains using the combined normalization and segmentation procedure implemented in SPM5, the latest version of SPM.

A MATLAB script for performing the enantiomorphic method is available from the authors.

## Footnotes

1 Note that since the displacement values are positive real numbers the mean, rather than the root mean square would suffice. However, we stick with RMS for the purposes of direct comparison with the only other paper to address this issue (Brett et al., 2001).
