# Supplementary material for: Enantiomorphic normalization of focally lesioned brains
Source: Neuroimage. 2008 Feb 1;39(3-3):1215–26. doi: 10.1016/j.neuroimage.2007.10.002 (PMC2658465; doi:10.1016/j.neuroimage.2007.10.002)
Supplement: Supplementary file 1 [file mmc1.pdf]

Enantiomorphic Normalization of Focally Lesioned Brains

Supplementary material

Lesion signal properties

The degree of distortion produced by a lesion will depend on how the signal within it differs from the normal case. Since lesions vary widely in this respect – ranging from CSF intensity in conventional imaging to unphysiologically high intensity on diffusion-weighted imaging – it is difficult to evaluate the entire spectrum of possibilities. Moreover, lesions will vary not only in signal intensity but also in the pattern of change in signal across the lesion. This will also be highly variable – for example, consider a glioma with cystic change, an abscess, a haemorrhagically-transformed stroke, etc.

In any case, the value of exploring the entire range of lesion patterns and intensities – even if that were practicable – is moot since both cost-function masking and the enantiomorphic technique are relatively insensitive to the signal within the bulk of the lesion: this area is either masked or corrected. However, since normalization algorithms tend to smooth the image before deriving the normalization parameters, cost-function masking may be affected by the propagation of the lesion beyond its boundaries resulting from the smoothing step. We have seen that this effect alone cannot explain the superiority of the enantiomorphic method, because the difference in accuracy between the two methods remains largely unchanged even when using artificially "benign" lesions, filled-in with the mean of the normal tissue intensity within the area of the lesion.

Nonetheless, we have also run another analysis using the real lesion images in the dataset from Brett et al., 2001, kindly provided by Matthew Brett. We extracted the lesions (all are derived from high resolution, T1-weighted scans), artificially grafted them to our T1 dataset, and evaluated the normalization error in exactly the same way as in our other analyses. The lesions were cropped in places where they crossed the midline. The results are shown in Figure S1.

Combined cost-function masking and enantiomorphic correction

The enantiomorphic method is applicable only where a lesion does not cross the midline. In other situations one might combine it with cost function masking – enantiomorphically correcting the non-overlapping lesion areas and masking the rest. To evaluate this combined approach, we used the lesions from the original dataset to create a new set of lesion maps where *both* hemispheres are lesioned, with each lesion being within 10% of the size of the other, and the two lesions affecting homologous parts of the two hemispheres within 40-60% of their extent. We then compared the combined method with cost function masking, using the T2 source images in the original analysis. The results are shown in Figure S2. The combined method appears to be superior to cost function masking alone.

Correcting for asymmetry effects

Since our method depends on the symmetry between the hemispheres, the quality of registration will vary slightly with the extent to which that relation is violated. Theoretically, this effect may influence the statistics subsequently performed on the data, and therefore we ought to consider some ways of ameliorating it. It should be pointed out, however, that the enantiomorphic method is better than cost function masking even in brain areas that are known to be asymmetrical (see Figure 6 in main text). Thus a correction is not necessary, but it can potentially further improve the fidelity of the statistics.

There are two effects to consider. First, certain areas of the brain will exhibit greater asymmetry than others – *consistently* across subjects. In these areas the use of our technique may introduce noise, but should not introduce bias, since the effect is the same across subjects. Second, in certain areas the degree of asymmetry may vary with the experimental grouping of interest, and therefore act as a confound. In this situation our technique could theoretically cause a bias.

The extent to which the first problem is significant is hard to quantify as there will always be great differences in the quality of registration from one area of the brain to another. As inspection of the MNI 152 T1 template readily shows, the cortical regions are far more variable than (say) the basal ganglia – the differences are striking and likely to be vastly greater than the noise introduced by the enantiomorphic correction as these can never be greater than the (relatively very small) asymmetry differences. Introducing a correction for this error would therefore seem to be a vain exercise, but theoretically one could interpret the statistics overlaid by a symmetry map, and introduce the asymmetry at a specific location as a covariate if making statistical comparisons *between* different regions of interest.

The second problem can be solved by including an asymmetry *image* as a covariate of no interest in the SPM model. Thus, the design matrix has a different asymmetry covariate at each voxel depending on the asymmetry of that voxel in the given subject. This is possible using the Biological Parametric Mapping toolbox (http://www.fmri.wfubmc.edu/WFU_BPM/BPMdownload.htm). An asymmetry image can be generated for each subject by taking the midline-aligned structural image and taking the root square of the difference between that image and its mirrored version. This image can then be transformed into template space using the normalization parameters for transforming the source image. Such an asymmetry-corrected model will therefore take into account inter-individual differences in asymmetry in a voxel by voxel manner.

A

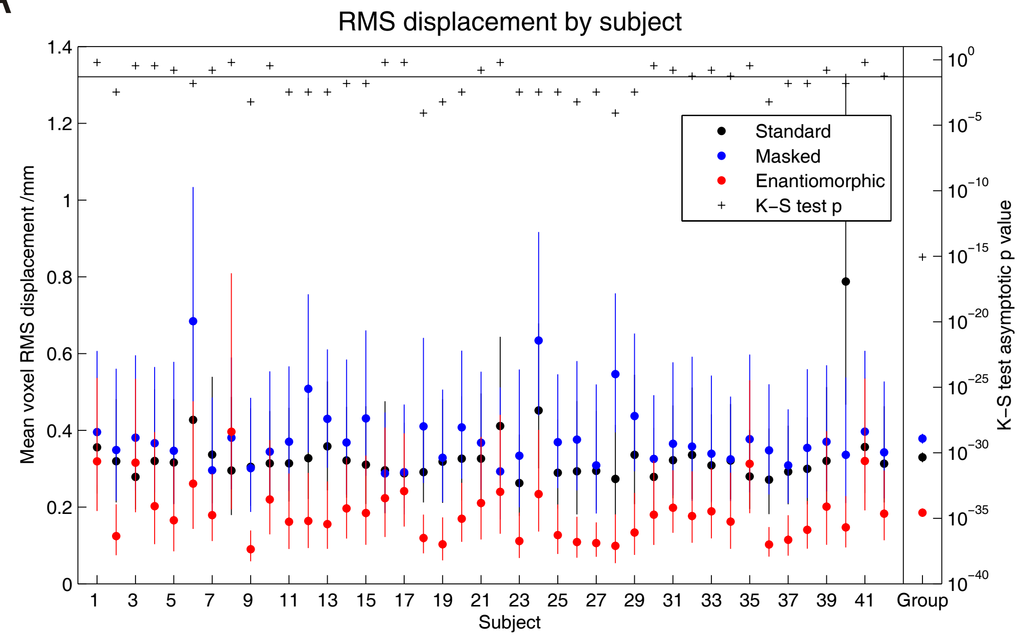

B

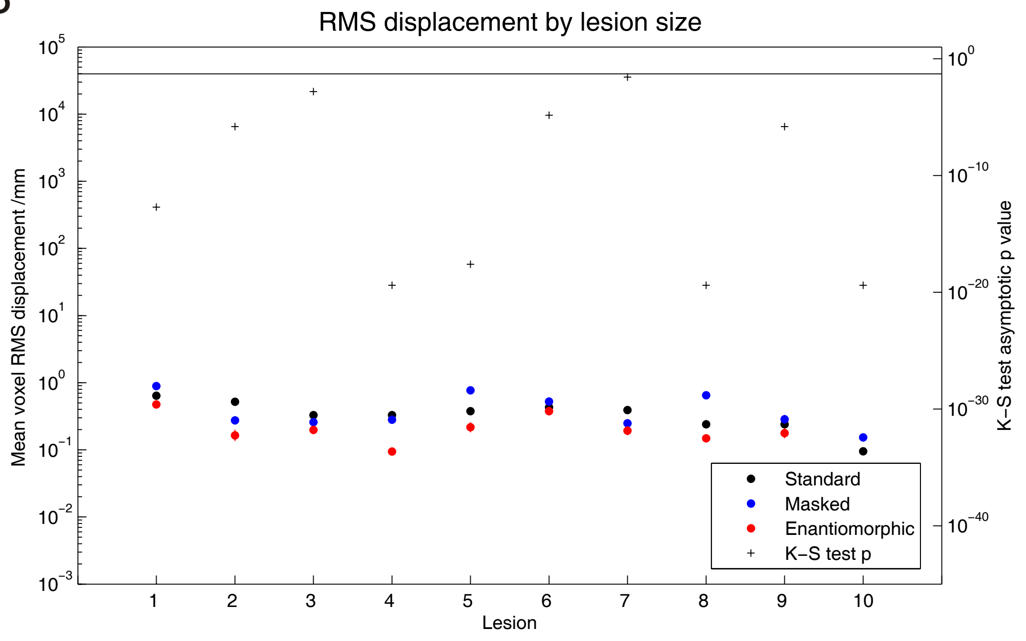

Figure S1. A. RMS displacement by subject (Brett et al., 2001 lesions). The left hand axis shows whole brain mean voxel RMS displacement with standard errors, plotted by individual subjects. The means include all 10 lesions and were derived – owing to log-normality – by first log-transforming the data, calculating the means and standard errors and anti-logging the results. The right hand side shows the asymptotic p values from a one-tailed, two sample Kolmogorov-Smirnov test carried out on the untransformed data from the masked and the enantiomorphic methods. The vertical line at the top defines the 0.05 significance level. The values in the far right column represent the group means and associated standard errors. The corresponding p value was derived from an appropriate K-S test on the group means.

B. RMS displacement by lesion size (Brett et al., 2001 lesions). The left hand axis shows whole brain mean voxel RMS displacement with standard errors, plotted by lesions size. The means include all 42 subjects and were derived – owing to log-normality – by first log-transforming the data, calculating the means and standard errors and anti-logging the results. The right hand side shows the asymptotic p values from a one-tailed, two sample Kolmogorov-Smirnov test carried out on the untransformed data from the masked and the enantiomorphic methods. The vertical line at the top defines the 0.05 significance level.

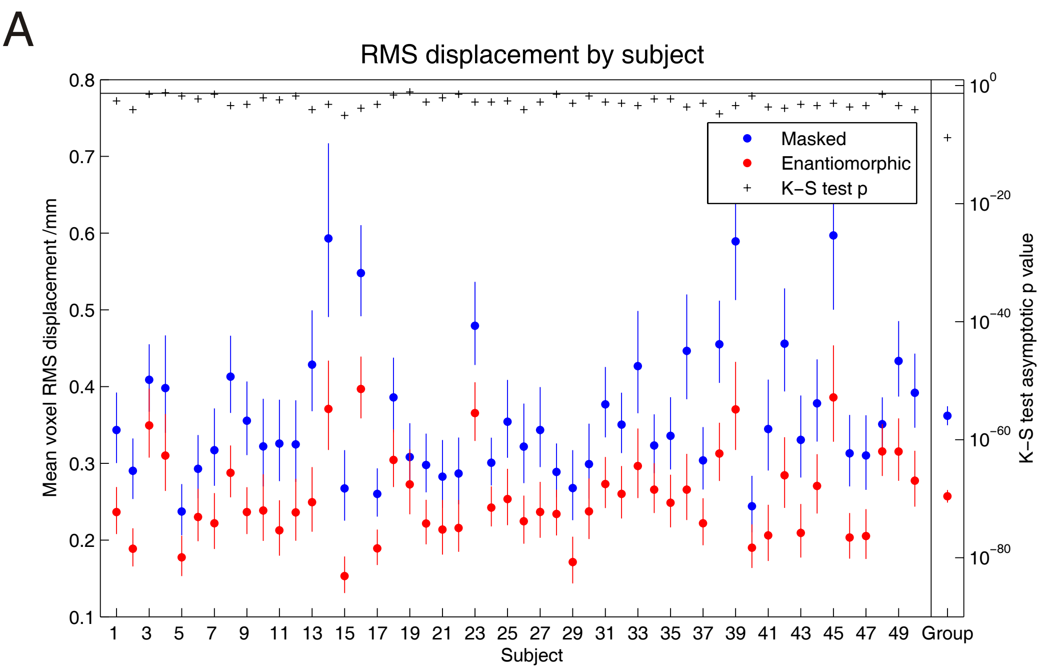

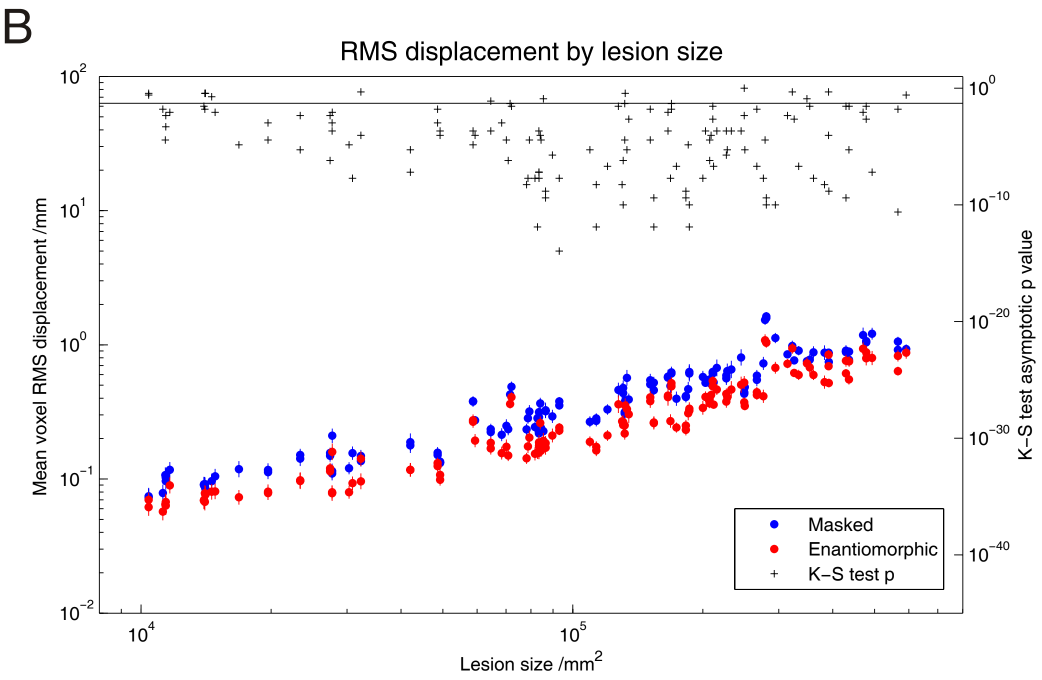

Figure S2. A. RMS displacement by subject (Combined method). The left hand axis shows whole brain mean voxel RMS displacement with standard errors, plotted by individual subjects. The means include all 305 lesions and were derived – owing to log-normality – by first log-transforming the data, calculating the means and standard errors and anti-logging the results. The right hand side shows the asymptotic p values from a one-tailed, two sample Kolmogorov-Smirnov test carried out on the untransformed data from the masked and the enantiomorphic methods. The vertical line at the top defines the 0.05 significance level. The values in the far right column represent the group means and associated standard errors. The corresponding p value was derived from an appropriate K-S test on the group means.

B. RMS displacement by lesion size (Combined method). The left hand axis shows whole brain mean voxel RMS displacement with standard errors, plotted by lesions size. The means include all 50 subjects and were derived – owing to log-normality – by first log-transforming the data, calculating the means and standard errors and anti-logging the results. The right hand side shows the asymptotic p values from a one-tailed, two sample Kolmogorov-Smirnov test carried out on the untransformed data from the masked and the enantiomorphic methods. The vertical line at the top defines the 0.05 significance level.
